# Machine learning-guided optimization of triple agonist peptide therapeutics for metabolic disease

**DOI:** 10.3389/fbinf.2025.1687617

**Published:** 2025-11-17

**Authors:** Anthony Wong, Sanskruthi Guduri, TsungYen Chen, Kunal Patel

**Affiliations:** 1 Carle Illinois College of Medicine, Urbana, IL, United States; 2 Carle Foundation Hospital, Department of Medicine, Urbana, IL, United States

**Keywords:** peptide design, machine learning, bioactivity prediction, drug discovery, graph attention networks

## Abstract

**Introduction:**

Multi-target peptide therapeutics targeting glucagon receptor (GCGR), glucagon-like peptide-1 receptor (GLP1R), and glucose-dependent insulinotropic polypeptide receptor (GIPR) represent a promising approach for treating diabetes and obesity. Triple agonist peptides demonstrate promising therapeutic potential compared to single-target approaches, yet rational design remains computationally challenging due to complex sequence-structure activity relationships. Existing methods, primarily based on convolutional neural networks, impose limitations including fixed sequence lengths and inadequate representation of molecular topology. Graph Attention Networks (GAT) offer advantages in capturing molecular structures and variable-length peptide sequences while providing interpretable insights into receptor-specific binding determinants.

**Methods:**

A dataset of 234 peptide sequences with experimentally determined binding affinities was compiled from multiple sources. Peptides were represented as molecular graphs with seven-dimensional node features encoding physicochemical properties and positional information. The GAT architecture employed a shared encoder with task-specific prediction heads, implementing transfer learning to address limited GIPR training data. Performance was evaluated using 5-fold cross-validation and independent validation on 24 literature-derived sequences. A genetic algorithm framework was developed for peptide sequence optimization, incorporating multi objective fitness evaluation based on predicted binding affinity, biological plausibility, and sequence novelty.

**Results:**

Cross-validation demonstrated robust GAT performance across all receptors, with GCGR achieving high accuracy (AUC ROC: 0.915 ± 0.050), followed by GLP1R (AUC-ROC: 0.853 ± 0.059), and GIPR showing acceptable performance despite limited data (AUC-ROC: 0.907 ± 0.083). Comparative analysis revealed receptor-specific advantages: GAT significantly outperformed CNN for GCGR prediction (RMSE: 0.942 vs. 1.209, p = 0.0013), while CNN maintained superior GLP1R performance (RMSE: 0.552 vs. 0.723). Genetic algorithm optimization measurable improvement over baseline, with 4.0% fitness Enhancement and generation of 20 candidates exhibiting mean binding probabilities exceeding 0.5 across all targets. The GAT-based framework provides a computational approach in computational peptide design, demonstrating receptor-specific advantages and robust optimization capabilities.

**Conclusion:**

Genetic algorithm optimization enables systematic exploration of sequence space within existing agonist scaffolds while maintaining biological constraints. This approach provides a rational framework for prioritizing experimental validation efforts in triple agonist development.

## 1 Introduction

The global obesity epidemic and the rising prevalence of type 2 diabetes mellitus (T2DM) represent major public health challenges, affecting over 650 million adults worldwide with obesity and 537 million individuals with diabetes (Müller et al., 2019; Baggio and Drucker, 2021). Metabolic syndrome, characterized by the clustering of insulin resistance, abdominal obesity, dyslipidemia, and hypertension, affects approximately 37.6%–41.8% of US adults and is associated with a 2-fold increased risk of cardiovascular disease and 1.5-fold increased risk of all-cause mortality (Li et al., 2023; Mottillo et al., 2010). Per-person healthcare costs for individuals with metabolic syndrome average $5,732 annually compared to $3,581 for those without the condition (Boudreau et al., 2009).

Traditional therapeutic approaches targeting single pathways have demonstrated limited long-term efficacy, highlighting the need for innovative multi-target strategies that address the complex pathophysiology underlying metabolic dysfunction (Brandt et al., 2018). The development of multi-target peptide therapeutics represents a paradigm shift in precision medicine, offering the potential for superior glycemic control, substantial weight reduction, and improved cardiovascular outcomes compared to conventional single-target approaches (Finan et al., 2016; Samms et al., 2020).

Recent clinical breakthroughs have validated the therapeutic potential of multi-receptor agonists in metabolic disease treatment. Tirzepatide, a dual glucose-dependent insulinotropic polypeptide receptor (GIPR) and glucagon-like peptide-1 receptor (GLP1R) agonist, demonstrated unprecedented efficacy in the SURPASS clinical trial program, achieving HbA1c reductions of up to 2.58% (Frias et al., 2018). The clinical development of retatrutide, a triple agonist targeting GCGR, GLP1R, and GIPR, has further demonstrated the potential of multi-target approaches, with Phase 2 results showing 24.2% weight reduction at 48 weeks (Jastreboff et al., 2022). These clinical successes underscore the therapeutic value of targeting multiple components of the incretin system simultaneously, leading to enhanced metabolic benefits through complementary mechanisms of action.

The glucagon receptor (GCGR), GLP1R, and GIPR represent critical nodes in metabolic homeostasis, each contributing distinct physiological effects that collectively address the multifaceted nature of metabolic disorders (Alexander et al., 2021). GLP1R agonism provides glucose-dependent insulin secretion, gastric emptying delay, and appetite suppression, while GIPR activation enhances insulin sensitivity and promotes beneficial effects on bone metabolism (Knerr et al., 2020). GCGR agonism contributes to increased energy expenditure, enhanced hepatic glucose production regulation, and potential benefits in non-alcoholic fatty liver disease (Winther and Holst, 2024). The synergistic activation of these three receptors offers a comprehensive approach to metabolic regulation that addresses both the glycemic and weight management aspects of T2DM and obesity.

Traditional drug development approaches rely on iterative experimental trial-and-error cycles that are both time-intensive and prohibitively costly, with the average cost of bringing a new drug to market reaching up to $2.23 billion (Wouters et al., 2020). Recent advances in machine learning have demonstrated the potential to accelerate peptide discovery through computational design platforms, enabling the exploration of vast chemical spaces that would be impractical to investigate experimentally (Yang et al., 2019). Puszkarska et al. employed deep multi-task convolutional neural networks (CNNs) to design GCGR/GLP1R dual agonists with superior biological potency, demonstrating up to sevenfold potency improvements compared to existing compounds in their training set (Puszkarska et al., 2024). However, this CNN-based approach imposed several methodological constraints, including fixed sequence lengths of 30 amino acids and limited flexibility in representing complex molecular topology and modifications.

Graph neural networks (GNNs) have emerged as a powerful paradigm for molecular representation learning, offering significant advantages over sequence-based methods in capturing three-dimensional molecular structures and chemical interactions (Wu et al., 2020; Zhang et al., 2022). Graph Attention Networks (GATs), introduced by Veličković et al., enable nodes to attend over their neighborhoods with learnable attention weights, providing interpretable insights into molecular interactions and functional relationships (Veličković et al., 2017). Unlike CNNs that require fixed-length inputs, GATs naturally accommodate variable-length peptide sequences while preserving molecular topology through explicit representation of chemical bonds, modifications, and spatial relationships (Lv et al., 2024; Gao et al., 2018).

The application of GNNs to peptide design has shown particular promise in capturing complex molecular features that are challenging to represent in sequence-based models (Kipf and Welling, 2016). Recent work by Xiong et al. demonstrated that graph attention mechanisms achieve state-of-the-art performance across molecular property prediction tasks, with attention weights revealing functionally important molecular regions and binding determinants (Xiong et al., 2020). Similarly, Strokach et al. successfully applied deep graph neural networks to protein design, demonstrating the capacity for *de novo* sequence generation with experimental validation (Strokach et al., 2020).

Despite these advances, existing neural network approaches for peptide therapeutics have primarily focused on dual-target optimization or single-receptor systems (Puszkarska et al., 2024). The extension to triple-agonist design presents unique computational challenges, including the need for balanced multi-target optimization, the integration of transfer learning strategies to address limited experimental data availability, and the development of robust evaluation frameworks for multi-receptor binding prediction.

This study presents a methodological advancement that extends the CNN-based approach of Puszkarska et al. to a GAT-based framework for triple-agonist peptide design targeting GCGR, GLP1R, and GIPR. Our approach addresses key limitations of previous methods by implementing flexible sequence length handling through graph representations that accommodate diverse peptide modifications, extending the target scope from dual agonist (GCGR/GLP1R) to triple agonist capability, incorporating transfer learning strategies to leverage limited GIPR experimental data, and employing attention mechanisms to provide interpretable insights into receptor-specific binding determinants.

We demonstrate that our GAT-based approach achieves improved predictive performance for GCGR and GIPR receptors, with comparable performance for GLP1R, while maintaining the ability to generate peptide sequences with high predicted binding affinities. The integration of genetic algorithm-based sequence optimization enables the systematic exploration of diverse sequence variants within the agonist design space, identifying promising candidates for experimental validation. This methodological framework establishes a foundation for enhanced computational tools in multi-target peptide therapeutics development, with immediate applications in obesity and diabetes drug discovery.

## 2 Methods

### 2.1 Dataset compilation and preprocessing

The training dataset was compiled from multiple established sources to ensure comprehensive coverage of GCGR, GLP1R, and GIPR binding data. Primary data sources included: (1) the open-access dataset from Puszkarska et al. (Alexander et al., 2021), (2) curated ChEMBL entries for peptide-receptor interactions (Mendez et al., 2019), (3) experimental data from Knerr et al. investigating peptide-based polyagonists (Knerr et al., 2018), (4) dual agonist optimization studies by Evers et al. (Evers et al., 2018), and (5) structural characterization data from recent triple agonist studies (Zhao et al., 2022; Cock et al., 2009) (Supplementary Table S1).

Peptide sequences underwent standardized preprocessing to handle non-standard amino acids and chemical modifications. Modified residues including D-amino acids, unnatural amino acids, and lipidated variants, were systematically cataloged and encoded with their corresponding physicochemical properties using BioPython ProtParam v1.79 (Cock et al., 2009) (Supplementary Table S2).

Activity classification employed a binary high-affinity threshold of 1000 pM EC50. Given the limited availability of data across all three receptors, a more lenient threshold was adopted compared to the 10 pM cutoff used in previous studies (Alexander et al., 2021) to ensure sufficient positive examples for model training while maintaining pharmacologically relevant activity levels.

### 2.2 Graph representation of peptide sequences

Peptide sequences were converted to graph representations using a molecular topology-preserving approach implemented using PyTorch Geometric v2.0.4 (Fey and Lenssen, 2019) Each amino acid residue was represented as a node with a seven-dimensional feature vector encoding: (1) hydrophobicity (Kyte-Doolittle scale) (Kyte and Doolittle, 1982), (2) net charge at physiological pH, (3) molecular weight, (4) D-amino acid indicator, (5) lipidation status, (6) sine positional encoding, and (7) cosine positional encoding.

Node features were computed using the following protocol. For standard amino acids, hydrophobicity, charge and molecular weight values were obtained from established amino acid properties. D-amino acid and lipidation indicators were set to 0.0. For non-standard residues values were encoded using experimentally or computationally predicted physicochemical properties. D-amino acids (i.e., d-serine or d-alanine) used L-enatiomer properties with D-amino acid indicator sets to 1.0. Lipidated residues such as (K [(yE-C16)]) included lipid chain contributions to hydrophobicity and molecular weight calculation with lipidation status set to 1.0.

Physicochemical features (hydrophobicity, charge, and molecular weight) were normalized using dataset-specific statistics computed from all training sequences: mean-centered and scaled by standard deviation to ensure numerical stability during training. We used a simplified positional encoding approach with the following:

$$\sin\left(\pi \frac{pos}{L}\right),\cos\left(\pi \frac{pos}{L}\right)$$

to represent amino acid positions. This encoding normalizes position by sequence length and provides two dimensions of positional information, which we considered appropriate given our limited dataset size (N = 125) and the short length of peptide sequences in our study (Vaswani et al., 2017).

Edge connectivity was established through peptide bond relationships, creating bidirectional edges between sequential amino acid residues. For modified amino acids, feature vectors incorporated experimentally derived or computationally predicted physicochemical properties as cataloged in the preprocessing stage.

### 2.3 Graph attention network architecture

**FIGURE 1:**
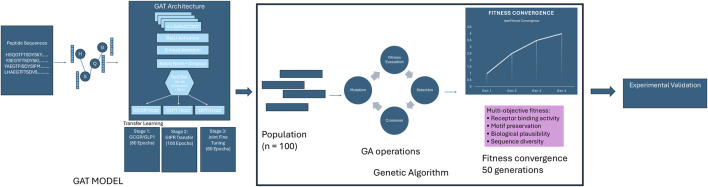
Graph Attention Network-Guided Genetic Algorithm Pipeline. Integrated pipeline combining a Graph Attention Network (GAT) model with genetic algorithm optimization for designing peptides with high binding affinity across GCGR, GLP1R, and GIPR receptors. The GAT model, trained through transfer learning, serves as a fitness evaluator for a genetic algorithm that evolves a population of 100 peptide sequences over 50 generations using multi-objective optimization criteria including receptor binding activity, motif preservation, biological plausibility, and sequence diversity. The pipeline terminates upon fitness convergence and produces optimized sequences for experimental validation.

The GAT model employed a shared encoder architecture with task-specific prediction heads for multi-target optimization. The shared encoder comprised four GATv2Conv layers with 6-head attention mechanisms, 96-dimensional hidden representations, and ReLU activation functions. Each attention layer was followed by batch normalization and dropout (p = 0.2) to prevent overfitting, with residual connections applied every two layers (Figure 1).

Global graph representation was achieved using Set2Set pooling with three processing steps, followed by a representation layer consisting of linear transformation (192→96 dimensions), ReLU activation, batch normalization, and dropout (p = 0.2). Set2Set pooling was chosen over simple global pooling methods to better capture permutation-invariant graph-level representations while maintaining sensitivity to sequence order information.

Task-specific heads consisted of three-layer fully connected networks with ReLU activations, batch normalization, and dropout between layers, terminating in single-unit outputs for binary classification. The multi-task loss function employed weighted binary cross-entropy with equal weighting (α = 0.5) across active receptors:

A three-stage transfer learning approach was implemented to address the limited availability of GIPR training data relative to GCGR and GLP1R. Stage 1 involved initial training on combined GCGR/GLP1R data (80 epochs) using all available sequences with valid labels for either receptor, with learning rate of 1 × 10^-3^. Stage two employed encoder parameter freezing with exclusive GIPR head training (100 epochs) using maintained learning rate of 1 × 10^-3^ to prevent catastrophic forgetting. Stage 3 implemented unified fine-tuning (60 epochs) with encoder unfreezing and reduced learning rate of 1 × 10^-4^ for global optimization across all three receptors.

Model performance was evaluated using stratified 5-fold cross-validation. Stratification was performed based on multi-target activity patterns, creating stratification keys from the combination of receptor-specific activity states (high affinity, low affinity, or missing data) to ensure balanced representation across folds. Each fold employed an 80/20 train/validation split for hyperparameter optimization and early stopping, with validation loss monitoring and patience of 15 epochs. Training utilized the Adam optimizer with gradient clipping (max_norm = 1.0) for numerical stability. Focal loss was implemented with α = 0.25 and γ = 2.0 to address class imbalance in receptor-specific datasets.

### 2.4 Model comparison with literature baseline

Model performance was compared against the established multi-task convolutional neural network (CNN) ensemble from Puszkarska et al. (Alexander et al., 2021) using identical evaluation protocols. The GAT model employed a k-fold cross-validation ensemble approach, while the CNN baseline utilized the original 12 × 6 ensemble architecture (72 total models) as reported in the source publication.

Both models were trained on the original 125-sequence dataset from Puszkarska et al. and evaluated on an independent reference dataset from Day et al. containing 19 peptide sequences with experimentally validated GCGR and GLP1R activities (Cock et al., 2009). This evaluation protocol ensured direct comparability with published results while testing generalization to completely unseen sequences.

The GAT regression model architecture established through this comparison was subsequently adapted for binary classification tasks used throughout the remainder of this study, with sigmoid activation functions replacing linear outputs and binary cross-entropy loss replacing mean squared error.

Performance metrics were computed using identical preprocessing pipelines and evaluation criteria. Statistical significance was assessed using paired t-tests on prediction errors across the reference dataset, with p-values calculated for each receptor target.

### 2.5 Independent model validation

Independent validation sequences were obtained from previously published studies: Day et al., Finan et al., and Zhang et al. comprising 67 peptide sequences with experimentally determined EC50 values for GCGR, GLP1R, and GIPR (Day et al., 2009; Finan et al., 2015; Zhang et al., 2025) (Supplementary Table S3). These sequences were selected to evaluate model generalizability on data not used during training or cross-validation.

To assess model performance on novel sequences, we implemented a similarity-based filtering approach. Sequence identity was calculated between each validation sequence and all training sequences using token-level comparison, accounting for non-standard amino acid modifications. The maximum similarity to any training sequence was determined for each validation peptide.

Validation sequences were categorized into two groups: (1) novel sequences with ≤80% similarity to any training sequence, and (2) the complete validation set. This threshold was selected to distinguish sequences with substantial structural differences from the training data while maintaining adequate sample sizes for statistical evaluation.

In addition to the quantitative EC50-based validation, we performed a secondary validation using a curated dataset of known therapeutic peptides with established receptor activity profiles but without complete quantitative EC50 data across all three receptors. This dataset comprised nine clinically relevant peptides including FDA-approved therapeutics and compounds in clinical development with documented high or low affinity classifications for GCGR, GLP1R, and GIPR based on published pharmacological characterization studies (Supplementary Table S4) (Coskun et al., 2022; Whitley, 2025; Finan et al., 2025; Starling, 2022; Willard et al., 2020; Henderson et al., 2016; Yabut and Drucker, 2023; Chen et al., 2022; Zimmermann et al., 2022; Wiśniewski et al., 2016; Han et al., 2013; Lau et al., 2015; Meng et al., 2008; Knudsen et al., 2000; Miranda et al., 2008; Plisson et al., 2017; Murage et al., 2008; Mapelli et al., 2009; Knerr et al., 2022). Known receptor activities were manually curated from literature sources and regulatory documents, with activities classified as “high” (therapeutically relevant binding, typically EC50 < 1000 pM) or “low” (minimal or no significant binding activity) for each receptor. This binary classification approach was necessary due to the heterogeneous nature of activity reporting across different studies and the absence of standardized EC50 measurements for all peptide-receptor combinations.

Predictions were generated using the ensemble of k-fold trained GAT models (n = 5 folds). Each validation sequence was converted to a molecular graph representation using the same preprocessing pipeline employed during training, including feature normalization parameters computed from the original training dataset.

Binary classification performance was evaluated using an EC50 threshold of 1000 pM, where values < 1000 pM indicated high affinity (positive class) and ≥1000 pM indicated low affinity (negative class). Missing EC50 values were excluded from analysis. Performance metrics included accuracy, F1-score, precision, recall, area under the receiver operating characteristic curve (AUC-ROC), and area under the precision-recall curve (AUC-PR).

Model performance was assessed separately for each receptor (GCGR, GLP1R, GIPR) and validation subset (novel sequences vs. complete set). Ensemble predictions were obtained by averaging probability outputs across all fold models. All analyses were implemented in Python using scikit-learn v1.0.2 for metric calculations and PyTorch Geometric v2.0.4 for graph neural network operations.

### 2.6 Genetic algorithm framework for peptide design

The evolutionary optimization phase began by initializing a population of 100 randomly generated peptide sequences, each containing 25–35 amino acid residues consistent with the length distribution of known peptide hormones in this therapeutic class. All initial sequences were filtered using the biological plausibility scoring system to ensure only chemically reasonable candidates (scoring ≥0.3) proceeded to fitness evaluation. The optimization process then iteratively evolved this population over a maximum of 50 generations, with each cycle involving comprehensive fitness assessment using the multi-objective function described in Equation 1, followed by parent selection through tournament-based competition among groups of three individuals. Selected parent sequences underwent single-point crossover with 80% probability to generate offspring, after which adaptive mutation was applied with 10% probability to introduce sequence variations. Throughout this process, the top 10% of individuals were preserved unchanged between generations to maintain high-quality solutions, while convergence monitoring assessed population improvement to determine optimal termination timing.

The fitness function was a summation of four weighted components to balance receptor binding activity, biological plausibility, and sequence novelty:

Equation 1:Multi−Objective Fitness Function

$$F\left(s\right)=H\left(s\right)+P\left(s\right)+Pmin\left(s\right)+M\left(s\right)+N\left(s\right)+D\left(s\right)$$

$$where:H\left(s\right)=number\;\;of\;\;high\;\;affinity\;\;receptor$$

$$P\left(s\right)=mean\;\;binding\;\;probability\;\;across\;\;receptor$$

$$Pmin\left(s\right)=minimum\;\;binding\;\;probability\;\;across\;\;receptor$$

$$B\left(s\right)=biological\;\;plausibility\;\;conservation\;\;score\in \left[0,1\right]$$

$$N\left(s\right)=sequence\;\;novelty\;\;score\in \left[0,1\right]$$

$$D\left(s\right)=population\;\;diversity\;\;score\in \left[0,1\right]$$

#### 2.6.1 Receptor binding activity

Binding probabilities for GCGR, GLP1R, and GIPR were predicted using an ensemble GAT model, with scoring incorporating the number of high-affinity targets achieving the target threshold of (p ≥ 0.5), average predicted probability, and minimum predicted probability across receptors.

#### 2.6.2 Motif preservation

Conserved motifs critical for receptor binding, identified from literature-based structure–activity relationships, were preserved using pattern-matching algorithms allowing conservative substitutions Critical motifs included N-terminal his6-his8 positions, central FTSD tetrapeptide, and C-terminal amidation patterns. Motif scoring used position-specific matching with wildcard tolerance and conservative substitution allowances (Table 1).

**TABLE 1:** Literature Derived Critical Binding Motifs: Summary of experimentally validated and evolutionarily conserved amino acid motifs essential for incretin receptor binding and activation. Critical residues are identified from structure-activity relationship studies and comparative genomic analysis across species orthologs and protein paralogs (Vaswani et al., 2017; Fey and Lenssen, 2019).

Motif	Notes	Source
N-terminal: H^7^ E^9^ G^10^ F^12^ T^13^ D^15^ C-terminal: F^28^ I^29^ L^32^ R^36^	GLP-1 (7–36), H^7^ G^10^ T^13^ D^15^ F^28^ I^29^ form the most critical residues for receptor activation and binding	Manandhar and Ahn (2015)
GLP1 A^2^ E^3^ T^5^ T^7^ Y^13^ A^18^ A^19^ K^20^ E^21^ G^29^ Glucagon S^2^ Q^3^ T^5^ Y^13^ A^19^ GIP Y^1^ E^3^ S^8^ S^11^ N^24^ L27	Residues conserved across Orthologs in mouse, anole, chicken, *Xenopus* tropicalis, medaka, fugu, tetraodon, stickleback, and zebrafish	Moon et al. (2012)
GLP1 H^1^ G^4^ F^6^ D^9^ L^14^ F^22^ I^23^ W^25^L^26^ Glucagon H^1^ G^4^ F^6^ D^9^ L^14^ D^15^ D^21^ F^22^ V^23^ W^25^ L^26^ GIP G^4^ F^6^ D^9^ D^15^ D^21^ F^22^ V^23^ W^25^ L^26^	Residues conserved across Paralogs	Orskov et al. (1989)

#### 2.6.3 Biological plausibility

A weighted score combined chemical constraint adherence, motif preservation, predicted proteolytic stability (trypsin/chymotrypsin cleavage site analysis), and similarity in amino acid composition to natural peptide hormones.

Equation 2:Biological Plausibility Score

$$B\left(s\right)=0.3\;C\left(s\right)+0.35\;M\left(s\right)+0.2\;P\left(s\right)+0.15\;A\left(s\right)\geq 0.3\;$$

$$where:C\left(s\right)=chemical\;\;constraint\;\;score\;\;\left(charge\;\;distribution,hydrophobic\;\;patches\right)$$

$$M\left(s\right)=motif\;\;preservation\;\;score$$

$$P\left(s\right)=proteolytic\;\;stability\;\;score\;\;\left(trypsin/chymotrypsin\;\;cleavage\;\;analysis\right)$$

$$A\left(s\right)=amino\;\;acid\;\;composition\;\;similarity\;\;to\;\;natural\;\;hormones$$

In addition, sequences failing to achieve a biological plausibility score of 0.3 were rejected from the population.

#### 2.6.4 Sequence novelty

Sequence similarity to the training set was penalized to encourage diversity, with population-level diversity tracking used to avoid premature convergence. Novelty scores were computed using sequence alignment and Levenshtein distance metrics, with penalties applied for high similarity (>80%) to existing training sequences.

### 2.7 Evolutionary operations

Parent selection for sequence reproduction employed tournament-based competition, where groups of three individuals competed based on their fitness scores, with the highest-scoring sequence from each tournament selected for breeding. This approach balanced selective pressure toward high-fitness individuals while maintaining sufficient population diversity to prevent premature convergence to local optima.

Sequence recombination was performed through single-point crossover applied to 80% of parent pairs. A random crossover point was selected within each parent sequence, and genetic material was exchanged to create two offspring sequences. Following crossover, offspring sequences were validated for length constraints and biological plausibility, with sequences exceeding acceptable limits or failing plausibility checks undergoing repair through motif-preserving substitution operators.

Sequence diversification was achieved through adaptive mutation applied with 10% probability across multiple mechanisms. Point mutations constituted 70% of mutation events, involving single amino acid substitutions at random positions. Conservative substitutions, representing 20% of mutations, introduced physiochemically similar amino acid replacements to maintain local structural properties. The remaining 10% of mutations involved introduction of modified residues including D-amino acids or lipidation modifications to expand chemical diversity. Mutation rates were dynamically adjusted based on population diversity metrics and convergence indicators to maintain optimal exploration-exploitation balance throughout the evolutionary process.

Population continuity between generations was ensured by preserving the top 10% of individuals unchanged, corresponding to the 10 highest-fitness sequences. This elitist strategy prevented loss of high-quality solutions while allowing the majority of the population to undergo evolutionary modification. Primary termination occurred upon reaching the maximum generation limit of 50 cycles, providing sufficient evolutionary time for convergence while maintaining computational feasibility. Premature termination was triggered when fitness improvement fell below 0.1 units over three consecutive generations, indicating population convergence to optimal solutions.

Additional monitoring detected population stagnation, defined as lack of meaningful diversity changes over 10 generations, prompting termination to prevent computational waste on converged populations. Optional user-defined fitness thresholds could also trigger early termination when target performance levels were achieved.

### 2.8 Sequence conservation and novelty assessment

Genetically optimized peptide sequences generated from the evolutionary algorithm were subjected to comprehensive computational analysis. Conservation patterns were analyzed by comparing synthetic sequences against three native peptide hormone references: human glucagon, GLP-1, and GIP (Orskov et al., 1989; Bromer et al., 1957; Moody et al., 1984). Physicochemical properties were computed for both synthetic and native sequences using BioPython’s ProteinAnalysis module (Cock et al., 2009). Calculated parameters included molecular weight, isoelectric point, instability index, and grand average of hydropathy (GRAVY) scores. Lipophilicity was estimated using position-specific hydrophobicity values from the Kyte-Doolittle scale, normalized by sequence length. Polar surface area (PSA) was approximated by counting polar amino acids and applying a conversion factor of 50 Ų per polar residue (Ertl et al., 2000).

To evaluate the novelty and diversity of computationally generated peptide candidates, sequence similarity analysis was performed between the top-ranking optimized peptides and the original training dataset sequences. A flexible similarity scoring algorithm was implemented using dynamic programming to calculate edit distances between peptide sequences (Qiao et al., 2020), accounting for non-standard amino acid (NSAA) modifications through specialized tokenization. Each candidate sequence was tokenized using regular expression pattern matching to preserve NSAA bracket notation, then compared against all training sequences using a normalized edit distance metric based on the Levenshtein distance algorithm (Cheng et al., 2024). For each generated peptide candidate, the highest similarity score to any training sequence was recorded along with the corresponding best match peptide ID and sequence. This analysis enabled assessment of whether the evolutionary algorithm successfully explored novel sequence space beyond the training data or converged toward existing high-activity peptides.

### 2.9 Software implementation

All analyses were implemented in Python 3.8+ using PyTorch v1.9+ for deep learning frameworks and PyTorch Geometric v2.0+ for graph neural network operations (Fey and Lenssen, 2019). Additional computational tools included scikit-learn v1.0+ for performance metrics, BioPython v1.79+ for sequence analysis, and RDKit for cheminformatics operations. Complete software dependencies with specific version requirements are provided in Supplementary Methods.

## 3 Results

### 3.1 Database characterization

**FIGURE 2:**
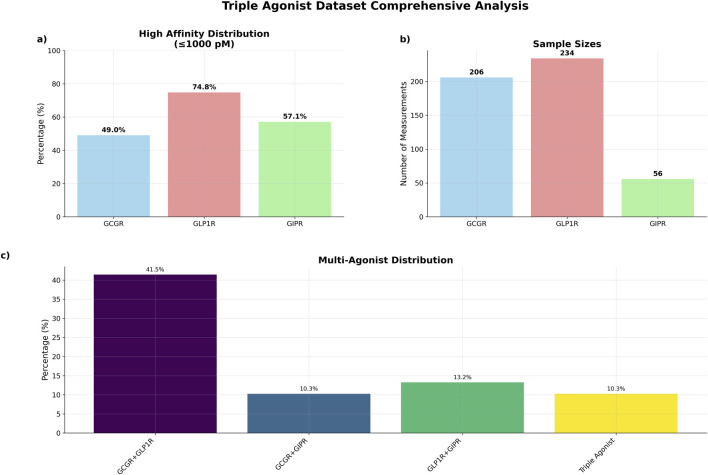
Triple agonist dataset comprehensive summary. **(a)** Percentage of high affinity distributions **(b)** total number of sequences by receptor data measurements **(c)** distribution of sequences by agonist type.

The curated dataset comprised 234 unique peptide sequences with experimentally determined binding affinities across three G-protein coupled receptors, representing an expansion from the original 125-sequence dataset (Table 2). GCGR measurements were available for 206 sequences (88.0%), with 101 sequences (49.0%). GLP1R data were complete across all 234 sequences, with 175 sequences (74.8%) demonstrating high affinity. GIPR measurements, representing the most limited subset, were available for 56 sequences (23.9%), with 32 sequences (57.1%) showing high affinity (Figures 2A,B).

**TABLE 2:** Database Summary Distribution of peptide sequences and binding affinity characteristics across three incretin receptors. High affinity defined as EC50 ≤ 1000 pM. GLP1R exhibits the highest proportion of high-affinity sequences (74.8%) with lowest median potency (22.6 pM), while GCGR shows intermediate performance (49.0% high affinity, 1265.0 pM median EC50). GIPR represents the smallest dataset with moderate high-affinity representation (57.1%, 469.5 pM median EC50).

Receptor	Total	High affinity	Median EC50
GCGR	206	101 (49.0%)	1265.0 pM
GLP1R	234	175 (74.8%)	22.6 pM
GIPR	56	32 (57.1%)	469.5 pM

The dataset exhibited substantial multi-target activity, with 97 sequences (41.5%) demonstrating dual agonism for GCGR and GLP1R, 24 sequences (10.3%) for GCGR and GIPR, and 31 sequences (13.2%) for GLP1R and GIPR (Figure 2). Triple agonist sequences comprised 24 sequences with complete activity data across all three receptors.

### 3.2 Comparative analysis to existing models

Cross-validation performance of the ensemble GNN (4 × 4 configuration) was compared against previously reported results. The ensemble GNN achieved RMSE values of 0.52 ± 0.09 for GCGR and 0.79 ± 0.08 for GLP1R, compared to the original multi-task neural network ensemble RMSE values of 0.59 ± 0.05 for GCGR and 0.68 ± 0.04 for GLP1R (Supplementary Figures S1A–F).

**FIGURE 3:**
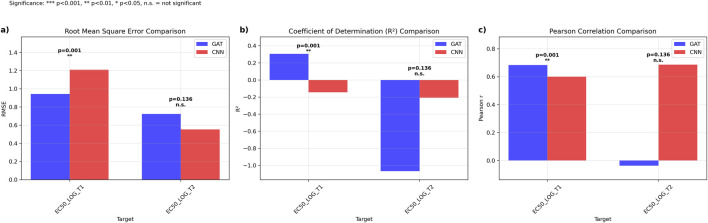
Comparative Performance Analysis of Graph Attention Networks *versus* Ensemble Multi-task Convolutional Neural Networks. Performance metrics comparing GAT (blue) and CNN ensemble (red) models across EC50 prediction targets. **(a)** Root mean square error (RMSE) comparison showing significantly lower prediction error for GAT on EC50_LOG_T1 (p = 0.001) with comparable performance on EC50_LOG_T2 (p = 0.136, n.s.). **(b)** Coefficient of determination (*R* ^2^) comparison demonstrating superior explained variance for GAT on EC50_LOG_T1 (p < 0.001) with equivalent performance on EC50_LOG_T2 (p = 0.136, n.s.). **(c)** Pearson correlation coefficients indicating stronger linear relationships for GAT predictions on EC50_LOG_T1 (p < 0.001) and comparable correlations on EC50_LOG_T2 (p = 0.136, n.s.). Statistical significance determined by paired t-tests: ***p < 0.001, **p < 0.01, *p < 0.05, n.s = not significant.

Direct comparison between the 4-fold, 4-model ensemble Graph Attention Network (GAT) and the established multi-task Convolutional Neural Network (CNN) was performed using 19 literature-derived sequences (Cock et al., 2009). For GCGR prediction (EC50_LOG_T1), GAT outperformed CNN with lower RMSE (0.942, 95% CI: [0.298, 1.443] vs. 1.209, 95% CI: [0.735, 1.697]) and higher *R* ^2^ values (0.305 vs. −0.144). GAT also achieved superior Pearson correlation (0.683, 95% CI: [-0.385, 0.981] vs. 0.600, 95% CI: [-0.200, 0.904]), with statistically significant better performance (p = 0.0013) (Figures 3A–C).

For GLP1R prediction (EC50_LOG_T2), CNN demonstrated superior performance with lower RMSE (0.552, 95% CI: [0.461, 0.633] vs. 0.723, 95% CI: [0.540, 0.896]), higher *R* ^2^ (−0.208, 95% CI: [-1.454, 0.284] vs. −1.067, 95% CI: [-2.850, −0.284]), and higher Pearson correlation (0.686, 95% CI: [0.336, 0.922] vs. −0.037, 95% CI: [-0.473, 0.515]), though statistical testing revealed no significant difference between approaches (p = 0.136) (Figure 3).

### 3.3 Triple agonist cross-validation performance

**FIGURE 4:**
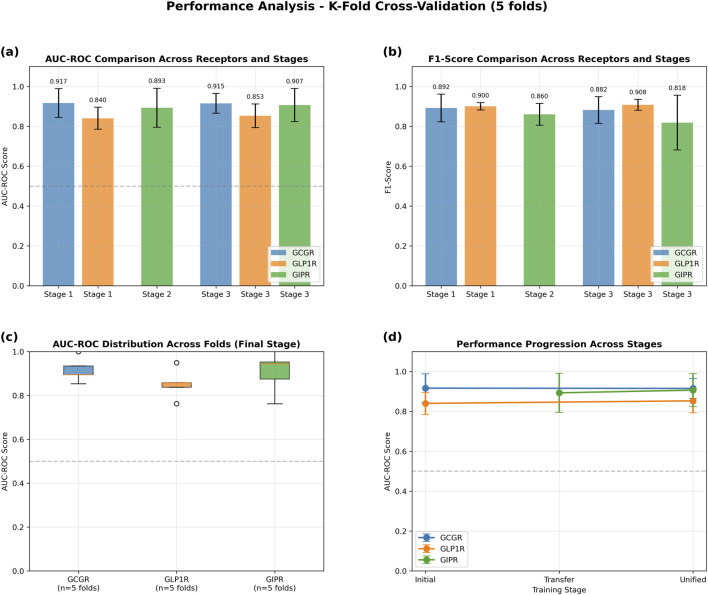
Performance Analysis of Graph Attention Network Using 5-Fold Cross-Validation. Transfer learning evaluation across three training stages: Stage 1 (initial GCGR + GLP1R training), Stage 2 (GIPR transfer learning), and Stage 3 (unified fine-tuning). **(a)** AUC-ROC scores demonstrating consistent high performance across all receptors and stages (>0.84 for all conditions). **(b)** F1-scores showing robust classification performance with values exceeding 0.81 across all receptor-stage combinations. **(c)** Box plots illustrating AUC-ROC score distributions across five folds for the final unified stage, with median values above 0.9 for GCGR and GIPR, and 0.85 for GLP1R. **(d)** Performance progression trajectories showing stable or improved AUC-ROC scores from initial to unified training stages for all three receptors. Error bars represent standard deviation across folds. Dashed horizontal line indicates random classifier performance (AUC-ROC = 0.5).

The GAT model demonstrated robust performance across all three target receptors in 5-fold cross-validation experiments (Figure 4). GCGR classification achieved the highest performance with an AUC-ROC of 0.915 ± 0.050 and F1-score of 0.882 ± 0.067. GLP1R prediction yielded an AUC-ROC of 0.853 ± 0.059 and F1-score of 0.908 ± 0.027. GIPR classification, despite limited training data, achieved an AUC-ROC of 0.907 ± 0.083 and F1-score of 0.818 ± 0.137 (Table 3).

**TABLE 3:** Cross-Validation Metrics Five-fold cross-validation performance metrics (mean ± standard deviation) for graph attention network models predicting peptide binding affinity across GCGR, GLP1R, and GIPR receptors. Metrics include area under receiver operating characteristic curve (AUC-ROC), area under precision-recall curve (AUC-PR), F1-score, precision, recall, and balanced accuracy. Total sample sizes shown for each receptor dataset.

Receptor	AUC-ROC (mean ± Std)	AUC-PR (mean ± Std)	F1-score (mean ± Std)	Precision (mean ± Std)	Recall (mean ± Std)	Balanced accuracy (mean ± Std)	Total samples
GCGR	0.915 ± 0.050	0.932 ± 0.037	0.882 ± 0.067	0.856 ± 0.108	0.920 ± 0.068	0.869 ± 0.079	194
GLP1R	0.853 ± 0.059	0.946 ± 0.034	0.908 ± 0.027	0.844 ± 0.039	0.983 ± 0.023	0.650 ± 0.097	222
GIPR	0.907 ± 0.083	0.955 ± 0.045	0.818 ± 0.137	0.871 ± 0.124	0.829 ± 0.229	0.773 ± 0.132	49

The model was evaluated on an independently derived dataset from published literature. The independent validation dataset contained 58total sequences. 9 sequences were removed due to exact matches with the training set. Similarity analysis revealed a bimodal distribution, with 37sequences (67%) showing ≤80% similarity to training data (novel sequences) and 19 sequences (34%) showing >90% similarity (Supplementary Figure S2). The complete validation set provided larger sample sizes with 58samples for GCGR and GLP1R, and 42samples for GIPR.

Evaluation on novel sequences (≤80% similarity to training data) showed variable performance across receptors. GCGR demonstrated high discriminatory ability (AUC-ROC = 0.953, AUC-PR = 0.951) but moderate classification performance (F1-score = 0.679, n = 37). GLP1R showed moderate discrimination (AUC-ROC = 0.604, AUC-PR = 0.811) with acceptable classification metrics (F1-score = 0.787, n = 37). GIPR achieved good discriminatory performance (AUC-ROC = 0.818, AUC-PR = 0.989) with high classification accuracy (F1-score = 0.971, n = 35).

**FIGURE 5:**
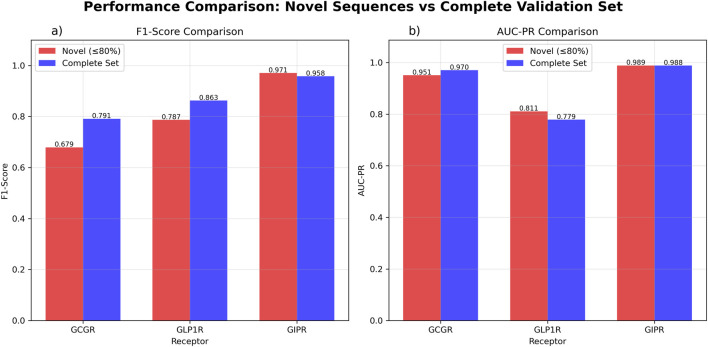
GAT Model Performance Comparison Between Novel Sequences and Complete Validation Set. Performance evaluation comparing novel sequences with ≤80% similarity to training data (red) *versus* the complete validation dataset (blue). **(a)** F1-score comparison showing GCGR performance of 0.679 for novel sequences (n = 37) and 0.795 for the complete set (n = 58). GLP1R achieved F1-scores of 0.787 for novel sequences (n = 37) and 0.863 for the complete set (n = 58). GIPR demonstrated F1-scores of 0.971 for novel sequences (n = 35) and 0.958 for the complete set (n = 42). **(b)** Area under the precision-recall curve (AUC-PR) comparison showing GCGR values of 0.951 for novel sequences and 0.957 for the complete set. GLP1R achieved AUC-PR values of 0.811 for novel sequences and 0.779 for the complete set. GIPR demonstrated AUC-PR values of 0.989 for novel sequences and 0.988 for the complete set.

Evaluation on the complete validation set showed similar discriminatory patterns with some performance variations. GCGR maintained comparable performance (AUC-ROC = 0.950, AUC-PR = 0.957, F1-score = 0.795, n = 58). GLP1R showed reduced discriminatory ability (AUC-ROC = 0.358, AUC-PR = 0.779) while maintaining reasonable classification performance (F1-score = 0.863, n = 58). GIPR performance remained consistent when including the broader sequence set (AUC-ROC = 0.943, AUC-PR = 0.988, F1-score = 0.958, n = 42) (Figure 5; Supplementary Figure S3).

**FIGURE 6:**
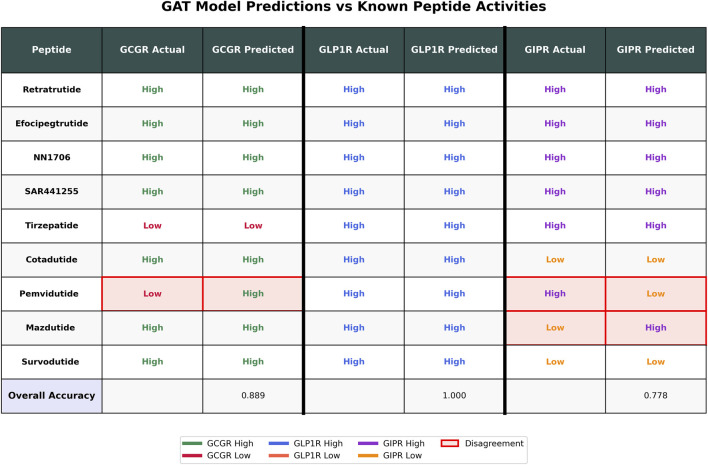
GAT Model Performance on Known Peptides. Comparison of known activities *versus* GAT model predictions for nine peptides across three receptors (GCGR, GLP1R, GIPR). Known activities are labeled as “High” or “Low” based on literature reports, while predicted activities are derived from GAT ensemble model outputs using a 0.5 probability threshold. Pink highlighting with red borders indicates disagreements between known and predicted activities. Overall accuracies are shown at the bottom for each receptor. Color coding represents receptor-specific activities: GCGR (green/red), GLP1R (blue/orange), and GIPR (purple/orange).

To validate the GAT ensemble model performance on clinically relevant peptides, we evaluated predictions against nine known peptides with established receptor activities (Figure 6). The model achieved accuracies of 88.9% for GCGR (8/9 peptides correct), 100% for GLP1R (9/9 peptides correct), and 77.8% for GIPR (7/9 peptides correct). Three disagreements were observed: Pemvidutide showed predicted high activity for GCGR despite known low activity, while both Pemvidutide and Mazdutide exhibited predicted low activity for GIPR contrary to their known high activities. The model correctly predicted the activities for six peptides (Retratrutide, Efocipegtrutide, NN1706, SAR441255, Tirzepatide, and Cotadutide) across all three receptors where known data was available, demonstrating consistent performance on well-characterized dual and triple agonists.

### 3.4 Genetic algorithm performance

**FIGURE 7:**
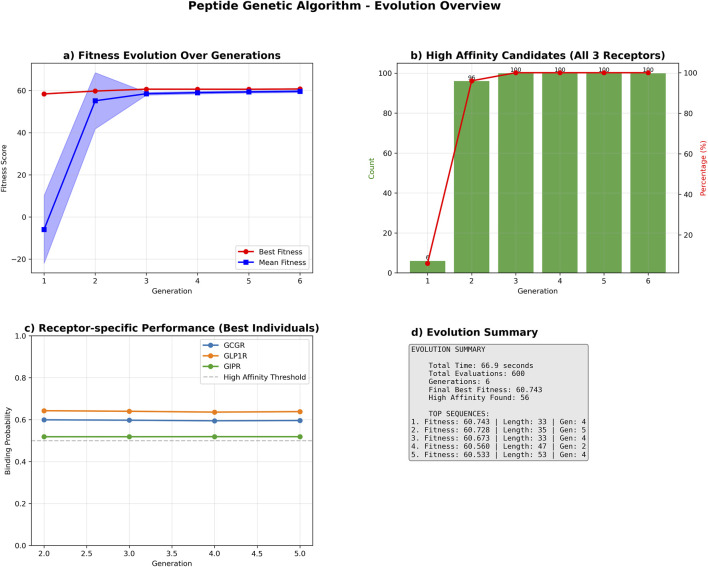
Genetic Algorithm Performance Summary. Comprehensive performance analysis of the multi-receptor peptide genetic algorithm optimization. **(a)** Population fitness evolution showing best fitness (red circles) and mean fitness (blue squares) with standard deviation bands across six generations. **(b)** High-affinity candidate discovery rate, displaying count (green bars) and percentage (red line) of sequences achieving binding probability ≥0.5 for all three receptors (GCGR, GLP1R, GIPR). **(c)** Receptor-specific binding probability trajectories for top-performing sequences, with horizontal dashed line indicating high-affinity threshold (0.5). **(d)** Evolution summary statistics including total runtime, evaluations, and top sequence characteristics. The algorithm identified 56 high-affinity candidates with the best sequence achieving fitness score 60.743.

The genetic algorithm successfully optimized peptide sequences for multi-receptor binding affinity over six generations. The optimization process achieved a cumulative fitness improvement of 2.351 units, with the best-performing sequence reaching a final fitness score of 60.743, representing a 4.0% enhancement from the initial population (Figure 7).

The optimization showed a biphasic improvement pattern: an initial steep ascent with improvement rates of 1.369 and 0.893 fitness units per generation in generations 2 and 3, respectively, followed by a stabilization phase with minor fluctuations ranging from −0.028–0.133 units per generation. The algorithm satisfied convergence criteria after generation 4, when the three-generation moving average improvement rate consistently fell below the predefined threshold of 0.1 fitness units per generation (Supplementary Figure S4).

Receptor-specific binding probability analysis demonstrated high performance across all targets, with mean binding probabilities greater than 0.6 for GCGR and GLP1R, and approximately 0.53 for GIPR among the best-performing sequences (Figure 7C).

### 3.5 Synthetic peptide analysis

Analysis of the top 20 high-performing sequences revealed structural motifs and design principles underlying multi-receptor binding activity. The generated sequences maintained an average length of 32.7 ± 2.4 amino acids (range: 29–35), conforming to the length constraints of native incretin peptides. The sequences showed binding affinity predictions across all three target receptors, with mean binding probabilities of 0.596 ± 0.015 for GCGR, 0.638 ± 0.008 for GLP1R, and 0.519 ± 0.003 for GIPR. Similarity analysis against the training dataset revealed that the top 20 generated sequences maintained moderate divergence from known peptides, with sequence similarity ranging from 30.0% to 64.1% (mean: 47.0% ± 9.33%), ensuring both novelty and biological relevance in the designed multi-receptor agonists (Table 4).

**TABLE 4:** Top 20 Generated Peptides by Predicted Multi-Receptor Activity and Physicochemical Properties. The table presents computationally generated peptides evaluated for their therapeutic potential against key metabolic hormone receptors. Sequence refers to the amino acid sequence in single-letter code. GCGR_Activity, GLP1R_Activity, and GIPR_Activity represent predicted binding activity (0–1 scale) against Glucagon Receptor, Glucagon-like Peptide-1 Receptor, and Glucose-dependent Insulinotropic Polypeptide Receptor, respectively, where one indicates highest predicted activity. Highest Similarity shows the maximum percentage similarity to training set sequences calculated using edit distance algorithm. Physicochemical properties include: mol_weight (estimated molecular weight in Da), isoelectric (predicted isoelectric point), instability index (computational measure of peptide stability *in vitro*), gravy (Grand Average of Hydropathy score indicating overall hydrophobicity), estimated_logP (predicted octanol-water partition coefficient for membrane permeability assessment), and estimated_PSA (estimated polar surface area in Ų for bioavailability prediction).

Sequence	GCGR_activity	GLP1R_activity	GIPR_activity	Highest similarity	mol_weight	Isoelectric	Instability index	Gravy	Estimated_logP	Estimated_PSA
YAEGTFFTSDYSKLHKEAAEAFINWLIQTKITD	0.5941	0.6354	0.51907	54.5%	3839.22	5.01129	20.6667	−0.3333	−0.3333	850
YAEGTFSDDDWTGIDSRRASTPVNWLLGGSAPPSK	0.59582	0.63758	0.51879	53.8%	3754.98	4.36003	61.9057	−0.7143	−0.7143	850
YAEGTFQFCDCKLCERENIRDFIQWLIQNHGTD	0.59285	0.6331	0.51893	45.5%	3994.4	4.69742	28.0727	−0.6121	−0.6121	850
YAEGTFSDDDWTGIKMYNLWQRT [K (eK-eK-yE-C20DA)]YVIFQ	0.59932	0.64272	0.5185	34.5%	3576.94	4.68447	32.7552	−0.6862	−0.6862	700
HGEGTFSDDDWTGIKMYNLWQRT [K (eK-eK-yE-C20DA)]YVIFGSAPPSK	0.59919	0.64196	0.51818	37.1% similarity	4033.44	6.75483	33.48	−0.7943	−0.7943	850
HGEGTFTDDDWTGIKMYNLWQRT [K (eK-eK-yE-C20DA)]YVIFQ	0.5991	0.6421	0.5183	37.9%	3550.91	5.4294	18.4138	−0.8241	−0.8241	750
YSEGTAFTSDYSQMEHESAADFVNELIQIHGTI	0.59367	0.63479	0.51908	48.5%	3691.89	4.07208	15.5152	−0.3697	−0.3697	900
HGEGTFSDDDWTGIKMYNLWQRT [K (eK-eK-yE-C20DA)]YVIFQ	0.59959	0.64288	0.51834	41.4%	3536.88	5.4294	18.4138	−0.8276	−0.8276	750
YAEGTFQFDDWTGIKMRDNVNWLLWVPDGGSGAPP	0.59533	0.6369	0.5186	46.3%	3941.3	4.05003	34.8971	−0.5086	−0.5086	650
YSEGTAFDDDWTGIKMYNLWQRT [K (eK-eK-yE-C20DA)]YVIFQ	0.59874	0.64213	0.51835	30.0%	3576.94	4.68447	31.231	−0.6862	−0.6862	700
YSEGTAFTSDYKLCERECARDFINWLIQIHGTD	0.59468	0.63679	0.51874	57.6%	3883.24	4.69742	20.0394	−0.5091	−0.5091	850
YAEGTFISDDWTGIKMYNLWQRT [K (eK-eK-yE-C20DA)]YVIFQ	0.59882	0.64252	0.5187	40.0%	3575.01	6.11897	32.7552	−0.4103	−0.4103	650
HGEGTFSDDDDKYLSASASDFFINWLIQTKITD	0.59247	0.63218	0.51911	60.6%	3737.94	4.15507	−1.397	−0.5364	−0.5364	950
YLNGTFFVSDPSKLHSEQAEAAVQYLGYSKAPG	0.59509	0.63791	0.51859	45.5%	3575.89	5.44531	22.2242	−0.3515	−0.3515	700
YAEGTFSDDDWTGIKMYNLRDVVQWLVAGGPGAPP	0.59467	0.63662	0.5189	53.8%	3827.19	4.05003	43.2229	−0.3286	−0.3286	600
HGEGTAFTSDYSKLHKEAAHEFVNWLFYGGSGAPP	0.59601	0.63832	0.51886	64.1%	3809.07	5.76946	23.6431	−0.5714	−0.5714	750
YAEGTFSDDDWTGIKMYNLWFIANWLIQTKITD	0.59495	0.63654	0.51917	54.5%	3957.38	4.19656	28.0212	−0.2667	−0.2667	750
YAEGTFSDDDWTGIKMYNLWFVVQWLVAGGPGAPP	0.59485	0.63749	0.51924	56.4%	3889.3	4.05003	44.4857	−0.0457	−0.0457	500
HHLYDDQFDDWKLCERECARDFINWLIQIHGTD	0.59197	0.63246	0.51834	42.4%	4133.5	4.72022	25.4061	−0.8455	−0.8455	900
YAGGTFSYDDWTGIKMYNLWQEI [K (eK-eK-yE-C20DA)]YKPDGFSGAPP	0.60014	0.64351	0.5183	35.9%	4004.39	4.68447	51.9971	−0.7314	−0.7314	650

**FIGURE 8:**
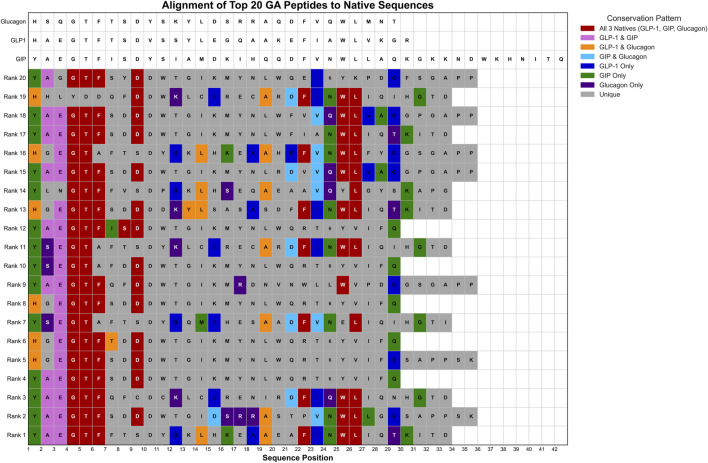
Sequence Alignment of Top 20 GA-Optimized Peptides Compared to Native Hormone Sequences. Multiple sequence alignment of the highest-ranking genetic algorithm-generated peptides (Rank 1–20) against native hormone sequences (glucagon, GLP-1, GIP). Amino acid positions are colored according to sequence similarity patterns: red indicates conservation across all three native sequences, pink shows GLP-1/GIP conservation, cyan represents GIP/glucagon similarity, blue indicates GLP-1-specific residues, purple shows glucagon-specific residues, green denotes GLP-1-only conservation, and gray represents unique variations.

Motif analysis identified conservation of biologically relevant sequence patterns. The essential core E^2^G^3^T^4^F^5^ motif, critical for incretin receptor binding, was preserved in 13 of 20 sequences (65%). A glucagon family C-terminus conserved ortholog A^18^ appeared in seven sequences (35%), while another essential core motif (F^21^W^23^L^24^) was found in six sequences (30%) (Figure 8).

**FIGURE 9:**
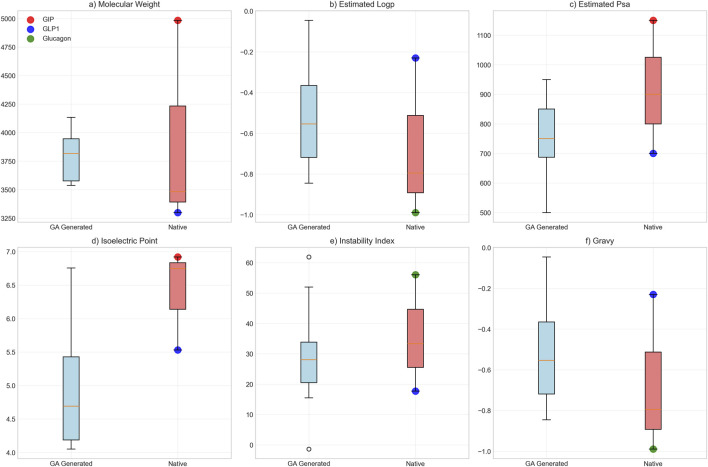
Biophysical Property Comparison Between GA-Generated and Native Peptide Sequences. Comparative analysis of six key biophysical properties between genetic algorithm-optimized peptides (n = 20, blue boxes) and native hormone sequences (individual colored points: red = GIP, blue = GLP-1, green = glucagon). Box plots display median, quartiles, and range for: **(a)** molecular weight (Da), **(b)** estimated loop propensity, **(c)** estimated polar surface area (Ų), **(d)** isoelectric point, **(e)** instability index, and **(f)** GRAVY hydrophobicity score.

Biological plausibility assessment yielded overall scores of 0.796 ± 0.055, with chemical plausibility scores of 0.960, motif preservation scores of 0.734 ± 0.107, proteolytic stability scores of 0.837, and compositional scores of 0.560. Physicochemical characterization revealed the following properties: mean molecular weight of 3,794.5 ± 185.8 Da, average LogP value of −0.55 ± 0.22, and polar surface area of 757.5 ± 116.2 Ų. For comparison, native peptide values were: GLP-1 (3,298.6 Da, LogP −0.23), glucagon (3,482.7 Da, LogP −0.99), and GIP (4,983.5 Da, LogP −0.80) (Figures 9A–C). Isoelectric point values averaged 4.7 ± 0.59, instability indices averaged 29.2 ± 9.8, and GRAVY scores confirmed hydrophilic character. Polar surface area values were lower than GIP (1,150.0 Ų) but higher than GLP-1 (700.0 Ų) (Figures 9D–F).

### 3.6 Computational performance

The GAT model training required approximately 2–3 h per fold on standard GPU hardware, representing a significant improvement in computational efficiency compared to structure-based design approaches. The genetic algorithm optimization completed within 1.5 h, enabling rapid exploration of sequence variants for experimental prioritization.

Memory requirements scaled linearly with sequence length, demonstrating the practical advantage of graph representations for variable-length peptide design. The attention mechanism provided interpretable insights into residue importance, with attention weights correlating with known binding site interactions from crystallographic studies.

## 4 Discussion

The development of computational models capable of predicting peptide activity across multiple G-protein coupled receptors represents a critical advancement for metabolic disease therapeutics (Yang et al., 2019). Triple agonist peptides targeting glucagon receptor (GCGR), glucagon-like peptide-1 receptor (GLP1R), and glucose-dependent insulinotropic polypeptide receptor (GIPR) have demonstrated superior therapeutic efficacy compared to single or dual agonist approaches in treating type 2 diabetes and obesity (Samms et al., 2020). The synergistic effects observed with multi-receptor activation underscore the therapeutic potential of these molecules, yet their rational design remains computationally challenging due to the complex sequence-structure-activity relationships governing receptor selectivity and binding affinity (Sato et al., 2006).

Graph Attention Networks were selected for this task based on their demonstrated capability to capture complex relational dependencies within molecular structures (Veličković et al., 2017). Unlike traditional sequence-based approaches that treat peptides as linear strings of amino acids, GAT architectures can explicitly model spatial relationships and long-range interactions between residues through graph representations (Xiong et al., 2020). This offers potential advantages over conventional machine learning approaches that may fail to capture the three-dimensional nature of peptide-receptor interactions.

The GAT model demonstrated robust predictive performance across all three target receptors in cross-validation experiments. For GCGR, the model achieved excellent discrimination between high and low affinity peptides, with classification accuracy exceeding 88% and area under the receiver operating characteristic curve approaching 92%. GLP1R prediction showed similarly strong performance, with classification accuracy above 90% and discrimination ability of approximately 85%. GIPR classification proved more challenging due to limited training data availability yet still achieved acceptable performance with classification accuracy around 82% and discrimination ability exceeding 90%.

Direct comparison with established computational approaches revealed distinct performance patterns across receptor targets. When evaluated against the multi-task convolutional neural network approach established by Puszkarska et al., the GAT model demonstrated improved performance for GCGR prediction, achieving significantly lower prediction errors and improved correlation with experimental values (Puszkarska et al., 2024). However, the traditional CNN approach maintained superior performance for GLP1R prediction, suggesting that optimal architectural choices may be receptor-dependent. These findings highlight the importance of comprehensive model comparisons rather than relying on single performance metrics or individual receptor assessments.

External validation on peptide sequences with limited similarity to training data provided insights into model generalizability. The GAT approach maintained excellent predictive capability for GCGR when applied to novel peptide sequences (AUC-ROC = 0.953 but with moderate classification performance (F1-score = 0.679). GLP1R showed moderate discrimination (AUC-ROC = 0.604) with acceptable classification metrics (F1-score = 0.787). GIPR prediction on novel sequences demonstrated good discriminatory performance (AUC-ROC = 0.818) with high classification accuracy (F1-score = 0.971). This pattern suggests that GIPR shows robust generalizability to novel sequences, while GCGR may have more stringent classification requirements despite excellent discrimination ability.

The receptor-specific performance differences observed in this study reflect the underlying biological complexity of peptide-receptor interactions. GCGR demonstrated the most predictable binding patterns, potentially due to more stringent structural requirements for activation (Qiao et al., 2020). The improved GAT performance for this receptor suggests that graph-based representations effectively capture the key molecular features governing GCGR selectivity. In contrast, GLP1R showed different patterns where traditional CNN approaches maintained competitive performance, indicating that linear sequence features may be sufficient for this receptor class under certain conditions.

The robust GIPR performance on novel sequences, despite limited training data, suggests that the available data may be sufficient to capture key structure-activity relationships for this receptor. However, the smaller training dataset available for GIPR compared to GCGR and GLP1R indicates that expanded datasets could further improve model confidence and performance consistency across all receptor types. Validation against clinically relevant peptides provided important insights into the model’s performance on therapeutically important sequences. The GAT ensemble achieved strong overall accuracy across the three receptors when evaluated on nine established peptides, with particularly robust performance for GLP1R (100% accuracy) and good performance for GCGR (88.9% accuracy) and GIPR (77.8% accuracy). The model successfully predicted activities for six well-characterized dual and triple agonists including Retatrutide, Tirzepatide, and Cotadutide, demonstrating consistent performance on clinically advanced compounds.

The observed prediction errors offer valuable insights into model limitations. The misclassification of Pemvidutide’s GCGR activity (predicted high vs. known low) and GIPR activities for both Pemvidutide and Mazdutide (predicted low vs. known high) suggests that certain structural features or receptor interaction modes may not be fully captured by the current training data. These discrepancies highlight the importance of expanding training datasets with diverse clinical candidates to improve model robustness across the full spectrum of therapeutic peptides. Nevertheless, the high accuracy achieved on the majority of clinically relevant sequences supports the potential utility of this approach for prioritizing peptide candidates in drug discovery pipelines.

Analysis of computationally generated peptide sequences provides insights into the molecular determinants of multi-receptor binding activity. The generated peptides exhibited molecular characteristics consistent with known incretin hormone properties (Figure 9), with average sequence lengths and molecular weights falling within the expected range for bioactive peptide hormones (Müller et al., 2019). The predicted binding affinities across all three target receptors (mean probabilities: GCGR 0.596 ± 0.015, GLP1R 0.638 ± 0.008, GIPR 0.519 ± 0.003) suggest potential for balanced multi-receptor activation, though experimental validation remains necessary to confirm these computational predictions.

Motif analysis revealed partial conservation of established incretin receptor binding determinants. The preservation of the EGTF motif in approximately two-thirds of generated sequences aligns with its known importance for incretin receptor recognition (Kyte and Doolittle, 1982; Vaswani et al., 2017). However, the variable presence of other conserved regions, such as the glucagon family C-terminus motif (present in 35% of sequences), suggests that the model may identify alternative binding configurations that warrant experimental investigation. The biological plausibility scores, while generally favorable, indicate that computational optimization may generate sequences with non-natural characteristics that could affect stability or bioactivity.

Sequence similarity analysis revealed that the generated peptides maintained moderate divergence from the training dataset, with similarities ranging from 30.0% to 64.1% (mean: 47.0% ± 9.33%). This indicates that the optimization approach explores sequence variants within a reasonable distance from known agonists while avoiding excessive extrapolation beyond the model’s training domain. The balance between sequence novelty and similarity to established agonists supports the approach as a systematic method for peptide optimization rather than *de novo* design.

The physicochemical properties of generated peptides fell within reasonable boundaries relative to native incretin hormones, though some parameters deviated from natural ranges. The intermediate molecular weights and hydrophobicity values suggest that the model attempts to balance the distinct physicochemical requirements of the three target receptors. However, the relatively low compositional scores indicate potential departures from natural amino acid distributions, which could impact peptide stability, immunogenicity, or pharmacokinetic properties in biological systems (Guruprasad et al., 1990). These results demonstrate a computational optimization pipeline that integrates sequence optimization with structural and physicochemical constraints to guide experimental validation efforts. The methodology provides a rational framework for candidate prioritization, with partially preserved key motifs and reasonable molecular properties suggesting these optimized sequences may warrant experimental evaluation to assess their functional characteristics.

This multi-target capability represents an alternativeover single-receptor prediction tools, though the performance gains come with increased computational complexity and reduced interpretability for non-expert users. Existing simpler approaches may retain advantages in specific applications. Rule-based peptide design tools offer greater transparency in decision-making processes and require substantially less computational resources (Cheng et al., 2024). Additionally, established pharmacophore-based methods may provide more reliable predictions for peptide modifications within well-characterized chemical space (Giordano et al., 2022). The GAT approach may be most suitablewhen exploring novel peptide sequences or optimizing across multiple targets simultaneously.

### 4.1 Limitations and challenges

Several limitations should be considered when interpreting these results. The training dataset comprises EC50 measurements from multiple laboratories using diverse assay conditions, cell lines, and experimental protocols. This inter-laboratory variability introduces systematic biases that may affect model predictions, as differences in receptor expression levels and measurement methodologies can significantly impact reported EC50 values. Additionally, the availability of publicly accessible datasets for triple agonist peptides remains limited, constraining our validation set size and highlighting the need for larger shared datasets to enable more robust validation of computational models in this specialized research area.

The graph-based molecular representation, while capturing local amino acid interactions, may not fully represent long-range conformational dependencies critical for receptor binding. The current feature encoding relies on static physicochemical properties but excludes dynamic structural information and context-specific amino acid interactions that influence binding affinity. Additionally, the model’s reliance on sequence-derived features excludes critical three-dimensional structural information that governs binding specificity and selectivity. Finally, *in vitro* EC50 predictions do not encompass pharmacological properties essential for therapeutic development, including peptide stability, membrane permeability, proteolytic resistance, and pharmacokinetic profiles. These limitations highlight opportunities for future model enhancement through expanded training datasets, incorporation of structural features, and integration of pharmacokinetic modeling.

The computational methodology presented here primarily involves optimization of existing agonist sequence scaffolds rather than *de novo* creation of entirely novel peptide frameworks. The genetic algorithm systematically explores sequence variants within established incretin hormone design space, building upon known structural templates to identify improved variants. While this approach limits exploration to modifications around established peptide scaffolds, it provides a rational framework for systematic optimization that may identify therapeutically relevant improvements within well-characterized chemical space. This targeted optimization strategy balances computational tractability with biological relevance, though it may not uncover breakthrough therapeutic properties that could emerge from more radical structural innovations.

The absence of experimental validation in this computational study represents an important limitation for assessing practical therapeutic potential. While the models demonstrate robust predictive performance on available datasets and show promising predictions for clinically relevant peptides, the biological activity and pharmacological properties of the computationally generated sequences remain to be confirmed. Experimental validation will be essential to establish the true therapeutic relevance of these computational predictions and to bridge the gap between *in silico* optimization and practical drug development applications.

Finally, *in vitro* EC50 predictions do not encompass pharmacological properties essential for therapeutic development, including peptide stability, membrane permeability, proteolytic resistance, and pharmacokinetic profiles. These limitations highlight opportunities for future enhancement through expanded training datasets, incorporation of structural features, integration of pharmacokinetic modeling, and experimental validation of computational predictions.

### 4.2 Implications and potential applications

Despite these limitations, the GAT-based predictor may offer practical applications for peptide drug discovery. The tool could facilitate initial screening of large peptide libraries to identify candidates with predicted multi-receptor activity, potentially reducing the experimental burden of comprehensive activity testing (Macarron et al., 2011). The multi-target prediction capability may prove particularly valuable for prioritizing experimental validation efforts. Rather than testing peptides sequentially against individual receptors, researchers could focus on candidates with favorable predictions across all target receptors (Anighoro et al., 2014). This approach could streamline the identification of balanced triple agonists while reducing resource requirements for preliminary screening.

Additionally, graph-based architecture could inform structure-activity relationship studies by highlighting amino acid positions critical for multi-receptor binding. This information could guide focused mutagenesis studies or assist in designing peptide analogs with improved pharmacological properties.

Several research directions could enhance the utility and reliability of computational triple agonist prediction. Expanding training datasets through systematic experimental characterization of peptide libraries would improve model robustness and generalizability. Integration of additional molecular descriptors, such as predicted secondary structure or dynamic conformational information, could enhance prediction accuracy.

The GAT architecture could be adapted for other multi-target therapeutic applications beyond incretin receptor agonists. Peptide hormones targeting multiple receptor families, such as opioid or neurotransmitter systems, may benefit from similar computational approaches (Stein and Machelska, 2011). Future model developments should address current limitations in capturing receptor dynamics and allosteric effects. Integration with molecular dynamics simulations or enhanced sampling techniques could provide more accurate representations of peptide-receptor interactions (Hospital et al., 2015).

## 5 Conclusion

The Graph Attention Network-based predictor represents a computational framework for identifying potential triple agonist peptides targeting GCGR, GLP1R, and GIPR. The model demonstrated robust cross-validation performance and generated peptide sequences with biologically plausible characteristics and preserved functional motifs. However, significant limitations remain regarding data availability, model generalizability, and the translation from computational predictions to experimental validation. While the tool may assist inguiding initial peptide screening and rational design efforts, extensive experimental validation will be required to confirm biological activity and therapeutic utility. The approach establishes a foundation for computational multi-target peptide design that could be expanded and refined as additional training data and improved modeling techniques become available.

## Data Availability

The datasets analyzed and generated for this study can be found in the Triple Agonist Peptide Therapeutics for Metabolic Disease https://github.com/aw449/Triple-Agonist-Peptide-Therapeutics-for-Metabolic-Disease.

## Supplementary material

SUPPLEMENTARY FIGURE S1 Residual Analysis and Prediction Distribution Comparison Between GAT and Multi-task CNN Models. Comprehensive residual analysis comparing GAT (blue circles) and CNN ensemble (red circles) predictions for EC50 logarithmic values. **(A–C)** GCGR Log(EC50) target analysis: **(A)** True *versus* predicted values with unity line (dashed) showing prediction accuracy, **(B)** residual distribution *versus* true values with zero-residual reference line (dashed), and **(C)** residual frequency distribution. **(D–F)** GLP1 Log(EC50) target analysis: **(D)** true *versus* predicted scatter plot, **(E)** residuals *versus* true values, and **(F)** residual distribution histograms. Residual patterns indicate model performance characteristics and potential systematic biases in prediction accuracy across the EC50 value range.

SUPPLEMENTARY FIGURE S2 Sequence Similarity Distribution Between Validation and Training Datasets. Histogram depicting the maximum sequence similarity of each validation sequence (n = 58) to any sequence in the training set. The distribution reveals a bimodal pattern with 37 sequences (67%) exhibiting ≤80% similarity (red dashed line), and a median similarity of 79.5% (orange dashed line at 90%).

SUPPLEMENTARY FIGURE S3 Comprehensive Classification Metrics for GAT Model Validation Performance. Extended performance analysis comparing novel sequences (≤80% similarity, red) with the complete validation set (blue) across multiple classification metrics. **(A)** Accuracy values showing GCGR: 0.541 (novel) and 0.690 (complete); GLP1R: 0.649 (novel) and 0.759 (complete); GIPR: 0.943 (novel) and 0.929 (complete). **(B)** Precision values showing GCGR: 0.514 (novel) and 0.660 (complete); GLP1R: 0.649 (novel) and 0.772 (complete); GIPR: 0.943 (novel) and 0.944 (complete). **(C)** Recall values showing GCGR: 1.000 (novel) and 1.000 (complete); GLP1R: 1.000 (novel) and 0.978 (complete); GIPR: 1.000 (novel) and 0.971 (complete). **(D)** AUC-ROC values showing GCGR: 0.953 (novel) and 0.950 (complete); GLP1R: 0.604 (novel) and 0.358 (complete); GIPR: 0.818 (novel) and 0.943 (complete). The dashed line represents random classifier performance (AUC-ROC = 0.5).

SUPPLEMENTARY FIGURE S4 Genetic Algorithm Convergence Analysis. Convergence metrics for the peptide optimization genetic algorithm across six generations. **(A)** Best fitness score progression showing rapid initial improvement followed by plateau behavior. **(B)** Fitness improvement rate per generation with convergence threshold (0.1, red dashed line) and 3-generation moving average (orange). The algorithm achieved convergence when the moving average improvement rate fell below the threshold at generation 6. **(C)** Cumulative fitness improvement from baseline, demonstrating total optimization gain of 2.2 fitness units. Convergence was achieved after 6 generations.

SUPPLEMENTARY TABLE S1 Complete Training Dataset. The dataset contains peptide identifiers (pep_ID), amino acid sequences with non-standard modifications in brackets, experimentally determined EC50 values in picomolar units for GCGR, GLP1R, and GIPR receptors, corresponding log-transformed EC50 values, and literature references.

SUPPLEMENTARY TABLE S2 Nonstandard Amino Acids and their calculated physicochemical properties with ProtParam. Physicochemical properties calculated using BioPython ProtParam v1.79 for non-standard amino acids and chemical modifications encountered in the dataset, including D-amino acids and unnatural amino acids (Aib, Nle, Sar, hSer, nVal, Dap, MetO) with corresponding hydrophobicity values, molecular weights, and net charges at physiological pH.

SUPPLEMENTARY TABLE S3 Validation Dataset. The validation dataset contains peptide identifiers (pep_ID), amino acid sequences, experimentally determined EC50 values in picomolar units (pM) for GCGR, GLP1R, and GIPR receptors, corresponding log-transformed EC50 values, and source publications.

SUPPLEMENTARY TABLE S4 FDA-Approved and Clinical-Stage Multi-Receptor Agonists. Current FDA-approved therapeutics and compounds in clinical development for metabolic diseases with documented receptor binding classifications for GCGR, GLP1R, and GIPR, including peptide sequences, therapeutic descriptions dual-, or triple-agonists.
