# Supplementary figures and images for: Machine learning-guided optimization of triple agonist peptide therapeutics for metabolic disease
Source: Front Bioinform. 2025 Nov 17;5:1687617. doi: 10.3389/fbinf.2025.1687617 (PMC12665757; doi:10.3389/fbinf.2025.1687617)

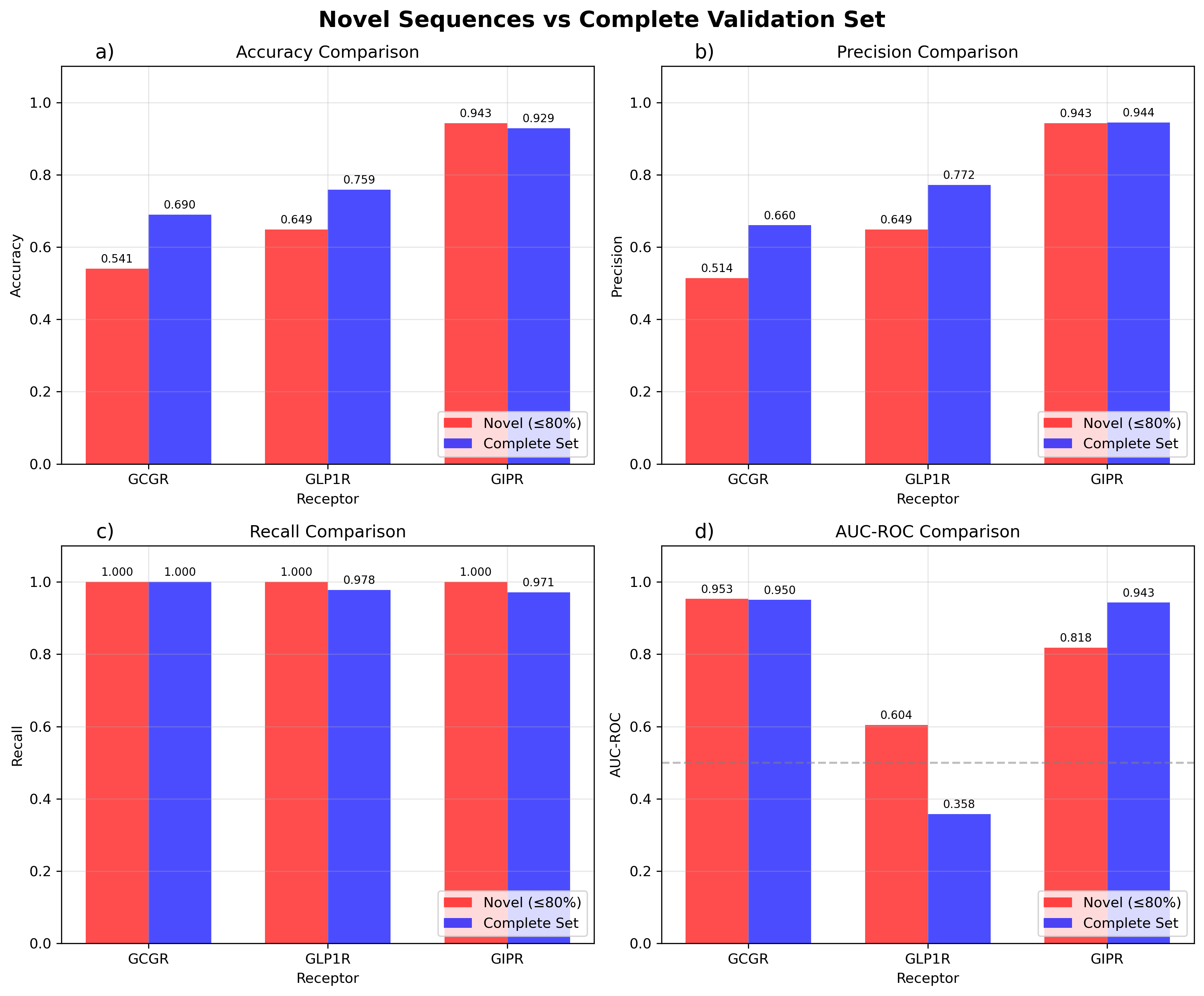

Supplement: Supplementary file 1 [file Image3.tiff]

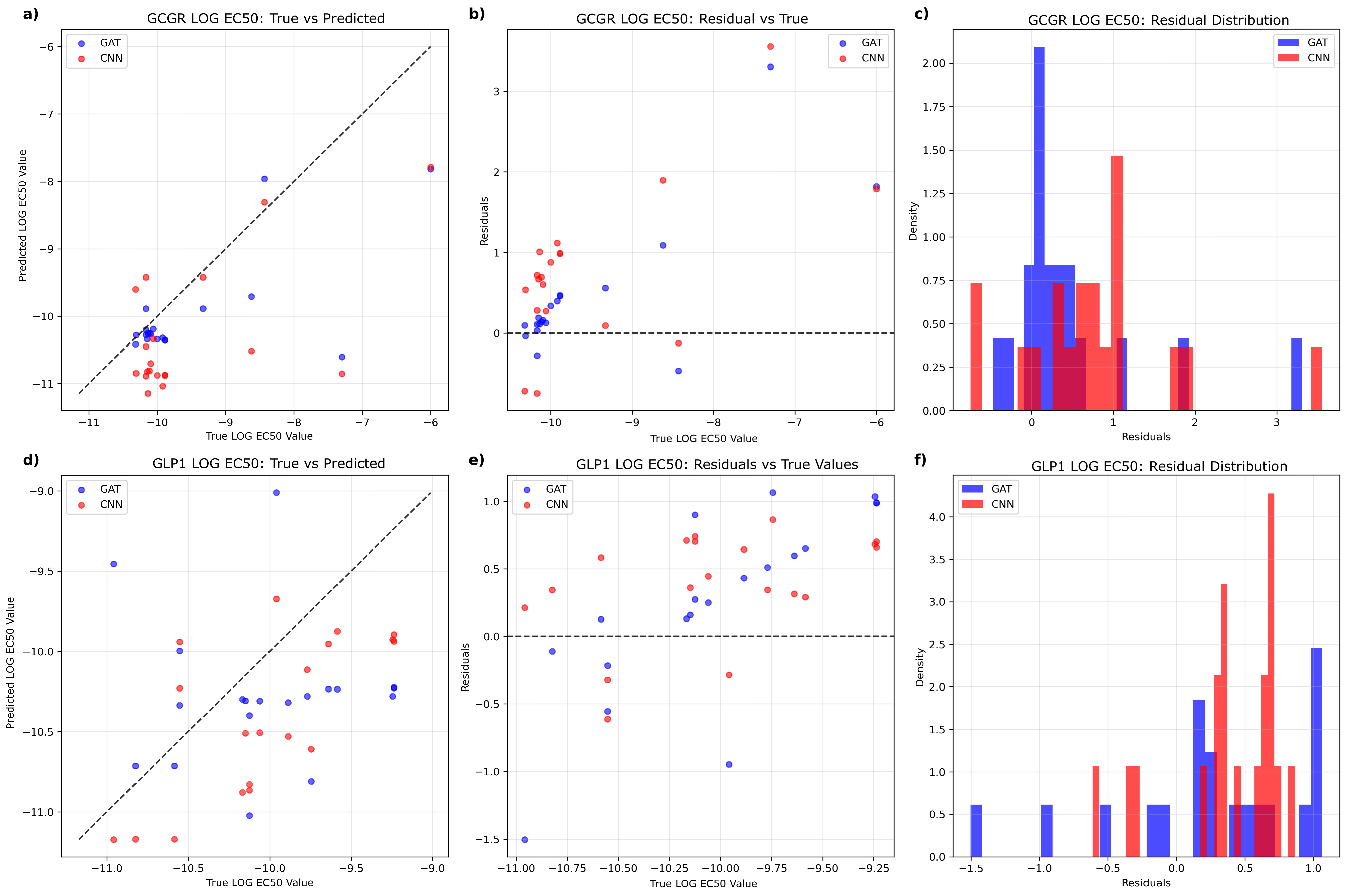

Supplement: Supplementary file 3 [file Image1.tiff]

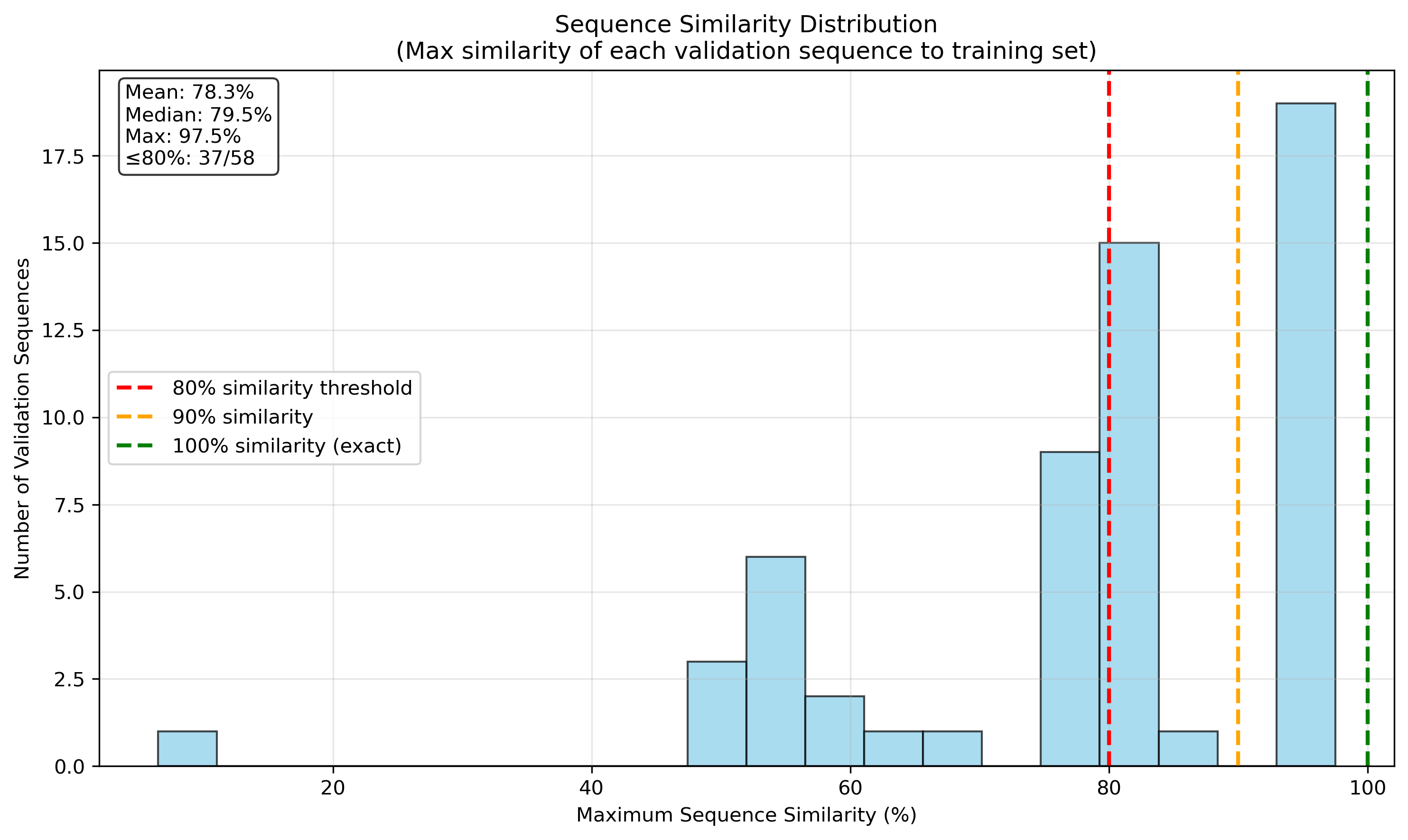

Supplement: Supplementary file 7 [file Image2.tiff]

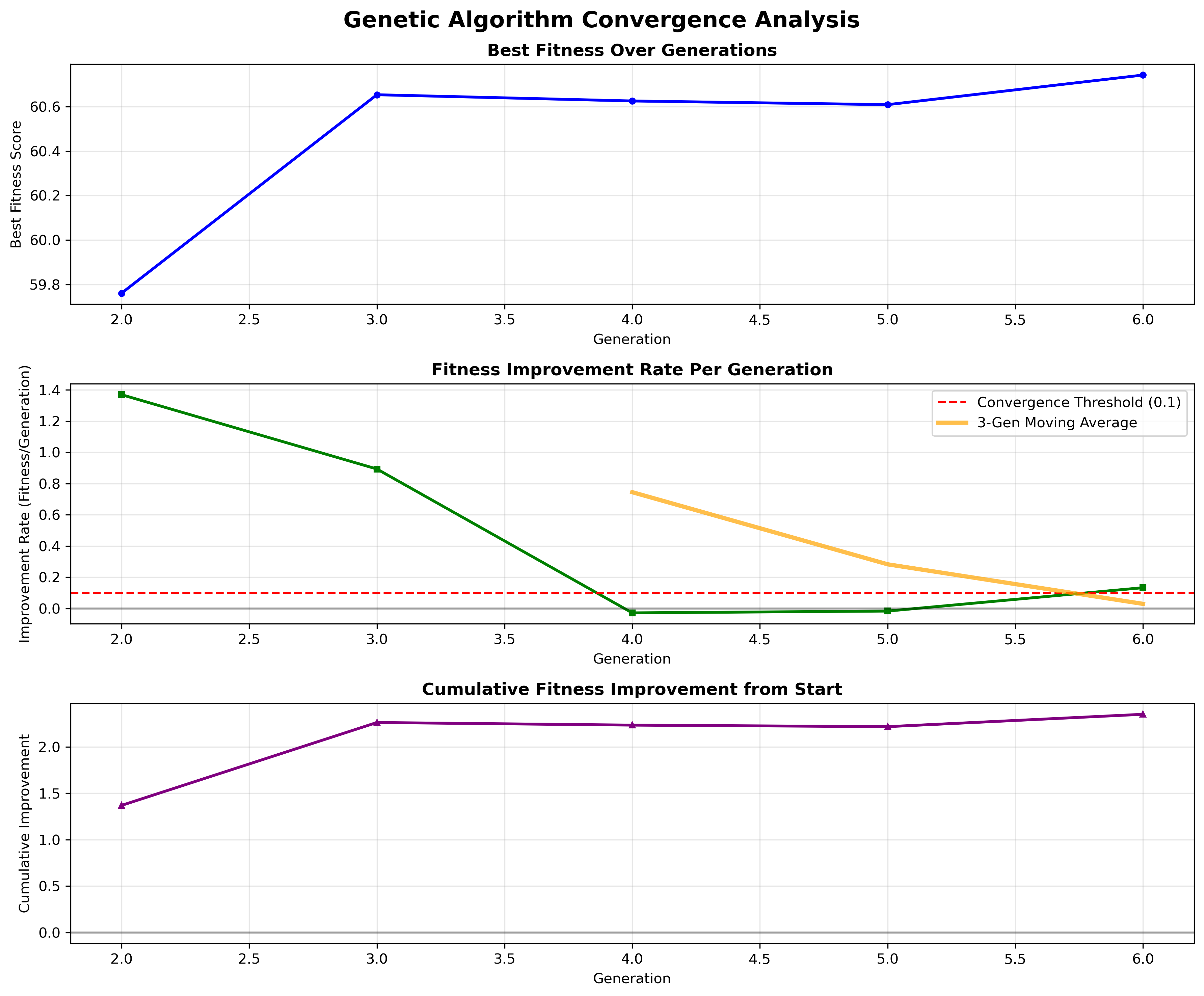

Supplement: Supplementary file 8 [file Image4.tiff]
